# Knowing me, knowing you: theory of mind in AI

**DOI:** 10.1017/S0033291720000835

**Published:** 2020-05

**Authors:** F. Cuzzolin, A. Morelli, B. Cîrstea, B. J. Sahakian

**Affiliations:** 1 School of Engineering, Computing and Mathematics, Oxford Brookes University, Oxford, UK; 2 Universita’ degli Studi Suor Orsola Benincasa, Naples, Italy; 3 Department of Psychiatry, University of Cambridge, Cambridge, UK

**Keywords:** AI ethics, artificial intelligence (AI), hot cognition, human–machine interaction, Theory of Mind

## Abstract

Artificial intelligence has dramatically changed the world as we know it, but is yet to fully embrace ‘hot’ cognition, i.e., the way an intelligent being's thinking is affected by their emotional state. Artificial intelligence encompassing hot cognition will not only usher in enhanced machine-human interactions, but will also promote a much needed ethical approach. Theory of Mind, the ability of the human mind to attribute mental states to others, is a key component of hot cognition. To endow machines with (limited) Theory of Mind capabilities, computer scientists will need to work closely with psychiatrists, psychologists and neuroscientists. They will need to develop new models, but also to formally define what problems need to be solved and how the results should be assessed.

Artificial intelligence (AI) has dramatically changed the world as we know it. Pivotal advances are associated with machine learning (ML), the branch of AI that deals with enabling machines to learn from data. A central role is played by deep learning (DL) (LeCun, Bengio, & Hinton, 2015), a technique based on artificial neural networks geared to efficiently support learning. Since DL has been coupled with reinforcement learning (RL, the notion that an agent learns by interacting with the real world and being rewarded or penalised accordingly), it has been shown to be capable of delivering, at times, superhuman performances. In a paramount example, the AlphaGo computer programme famously managed to consistently beat the world champion at Go, while delivering new insights on successful game strategies.

These ground-breaking achievements have generated overconfidence in AI and what it can achieve, especially in the short term. Venture capital is flocking to AI start-ups. People are expecting autonomous vehicles to appear on our roads at any moment. Some recent serious incidents, however, have raised red flags. Accidents involving prototype autonomous cars have led to casualties (Tesla Deaths, 2020), questioning how ready the technology really is for use in potentially dangerous situations, in which humans and machines closely interact. Strikingly, these accidents were caused by the vehicle failing to perform relatively simple tasks, such as object detection or obstacle tracking and avoidance (Hawkins, 2019).

In fact, far more sophisticated functions need to be in place before AI can be safely deployed in high-risk and potentially dangerous situations. Expectations of the current use of AI may need to be revised. Consider, for instance, autonomous driving. A smart car needs to make reliable predictions about human behaviour in real time, for example, in order to pre-emptively adjust speed and course to cope with a child's possible decision to abruptly cross the road in front of them. Deep neural networks can efficiently identify human actions in streaming videos, as motion patterns (Singh, Saha, Sapienza, Torr, & Cuzzolin, 2017). The latter, however, may be deceiving, as humans can suddenly change their minds based upon their own mental processes, thoughts and motivations, and things they see around them. In our example, children previously walking on the pavement towards school may spot an ice cream van across the road, and decide to dart across the road to get their ice cream. No predictive system functioning purely on past observed motion could be accurate and trustworthy enough in such complex environments, without taking into account context and the nature of the other agents involved. Human beings, on the other hand, can predict others' future behaviour even when no motion is present, just by quickly assessing the ‘type’ of person involved and the scene around them (e.g. an elderly person standing in a hallway is likely to decide to take the elevator, rather than the stairs).

This flags the need for AI to tackle ‘hot’ cognition, such as how a person's thinking is influenced by their emotional state (Lawrence, Clark, Labuzetta, Sahakian, & Vyakarnum, 2008; Taylor Tavares, Drevets, & Sahakian, 2003). Hot cognition refers to emotional and social cognition, including Theory of Mind (ToM). It contrasts with ‘cold’ cognition, in which the processing of information is independent of emotional involvement. Social cognition aims to understand social phenomena (i.e. the way people deal with other people) by investigating the underlying cognitive processes. With the emergence of artificial constructs able to show some (limited) intelligence, the concept needs to be extended to how humans deal with intelligent machines (e.g. an airline's conversational bot) and *vice-versa*.

## Theory of Mind

ToM is a major component of social cognition (Baron-Cohen, 1995). In cognitive psychology, the term refers to the set of processes and functions of the human mind that allow an individual to attribute mental states to others. For instance, looking at John inspecting the inside of the refrigerator, I can deduce that ‘John is hungry’. I may then stand up to offer him food that is in the refrigerator and some that is in the kitchen cupboard. The ability of putting yourself in somebody else's shoes is a crucial evolutionary advantage for humans, for it allows us to better interact with our environment and cooperate more effectively with our peers. The two dominant approaches to ToM are Theory-Theory (TT) and Simulation-Theory (ST; Harris, 1992). TT argues that children formulate theories which they then confirm or disprove through experience, just as a scientist would do. TT is problematic in its assumption that an individual generates a very large number of theories about other people and their behaviour, against the ‘cognitive economics’ principle which has been shown to characterise various mental functions. ST, instead, defends a simulation process which consists of taking someone else's perspective to understand their reasoning, while using many of the same cognitive mechanisms involved in making decisions from a first-person perspective. This is what leads to the ‘cognitive savings’ in the ‘cognitive economics’ principle. While some TT work is indeed concerned with cognitive economics (Gershman, Horvitz, & Tenenbaum, 2015) and computational efficiency (Baker, Jara-Ettinger, Saxe, & Tenenbaum, 2017; Pöppel & Kopp, 2018), the idea of ‘cognitive savings’ gained from sharing the first-person decision-making mechanisms appears to be less developed.

## A machine Theory of Mind

We claim that ToM needs to be incorporated into intelligent machines if they are to smoothly share environments built by human beings for human beings. To come back to our initial example, the main stumbling block towards the actual use of autonomous vehicles is safety. Safety relies on smart cars being able to understand and predict human behaviour. As argued above, naive pattern recognition is incapable of producing accurate predictions of complex and spontaneous human behaviours. The problem of anticipating human behaviour has in fact only recently risen to the attention of the computer vision community (Felsen, 2019), while, in contrast, it has been studied for a longer time by robotics researchers, especially in the context of human–robot interaction (Koppula & Saxena, 2016). What is truly required is an understanding of how humans reason, as well as their goals and motivations.

In terms of potential impact, ToM AI constructs could play a significant role in empathetic healthcare, for instance in interactions with individuals with neurological diseases, including Alzheimer's, and psychiatric disorders, such as depression, autism-spectrum disorder and schizophrenia. Such approaches could improve the efficacy of psychological treatments, such as cognitive behavioural therapy or mindfulness. In the longer term, we could imagine robotic companions for disabled people capable of understanding and projecting emotions. Work in this area would impact the current debate on moral AI, helping machines make ethical decisions in critical situations. As people realise machines are capable of reasoning like them, there will be greater trust in AI.

To date, however, AI has mostly focussed on ‘cold’ cognition, especially how to extract information from data. For example, machines are extremely good, and better than humans, at playing strategic games such as chess (in part because of their crushing advantage in terms of processing speed). In a medical context, they have proved able to diagnose diabetes from retinal images with higher accuracies than a human physician (De Fauw et al., 2018). Fruitful interactions between humans and machines are at the core of imitation learning (Ho & Ermon, 2016), a framework in which rewards are replaced by ‘tutorials’ given by humans to machines.

The flip side of the coin is that past efforts in incorporating ToM in machines have widely neglected the crucial learning aspect. Most relevant work has been done within the field of a multi-agent system, whereby an agent is an entity that autonomously tries to reach goals. Belief-Desire-Intention (BDI) models (Georgeff, Pell, Pollack, Tambe, & Wooldridge, 1998) have been proposed to emulate the functioning of the human mind, albeit in a simplistic way. Attempts have also been made to implement social norms that agents must follow, or simple ‘personality traits’. Studies have been conducted from theoretical perspectives as varied as Markov decision processes (Baker, Saxe, & Tenenbaum, 2011), multi-agent RL, evolutionary robotics or game theory (Yoshida, Dolan, & Friston, 2008).

The vast majority of these models, however, fail to reflect the real functioning of the human mind. A strong limitation of most of them is their inability to continuously and dynamically learn from experience and, thus, update the very rules of learning. Other agents' mental states are often assumed to be predetermined. Moreover, most of these models use reasoning processes based exclusively on propositional logic, which seems to reflect the way humans use language, but not the actual way the brain works, nor how knowledge is represented and organised within it.

## Theory of Mind *v.* brute-force learning

One potential criticism of the computational ToM approach is that, in some cases, learning from direct observations of other agents might be enough to predict their future behaviour, without trying to infer their mental states. However, considering once again the self-driving cars scenario, multiple reviews (Rasouli & Tsotsos, 2020; Rudenko et al., 2019) have highlighted the insufficiency of models which directly map observations to actions. Previous work on computational ToM (Baker et al., 2017) has also shown that ‘cue-based’ models, which go directly from observations to mental states via pure pattern recognition, perform worse than ToM models in terms of prediction accuracy.

Importantly, machine ToM approaches are potentially capable not only to predict the future behaviour of a person, but also to provide an explanation for the observed behaviour, linking, in a way, appearance and essence.

A sceptic might also argue that a pure (deep) learning approach might be sufficient for a spontaneous ToM to emerge. Indeed, prominent recent work (Rabinowitz et al., 2018) endorses a similar philosophy, casting computational ToM as a meta-learning problem. While the results achieved were impressive (most particularly, the authors' neural network learned to pass a ‘Sally-Anne’ like test, recognizing other agents' false beliefs; Wimmer & Perner, 1983), this required very large amounts of training data. Indeed, Jara-Ettinger (2019) points out that the experiments reported in Rabinowitz et al. (2018) required 32 million samples to learn to perform goal inference at a level similar to that of a 6-month-old infant. If infants learned ToM this way, 175 000 labelled demonstrations would be required every day during those 6 months.

More recent results cast doubt over the possibility of a pure learning-based approach for computational ToM for even more fundamental reasons. Jara-Ettinger (2019) has shown how ToM can be cast as inverse RL (IRL), a very popular RL framework for learning human preferences (or ‘reward functions’). Most IRL approaches assume some simplified form of human rationality. It has long been known, though, that humans consistently deviate from those assumptions (Tversky & Kahneman, 1975). Armstrong and Mindermann (2018) showed that, if no assumptions are made about human rationality, then a human's preferences cannot be recovered from behaviour only, no matter how much data the system can learn from. Even adopting principles similar to Occam's razor does not address the problem. It needs to be stressed that the impossibility result in Armstrong and Mindermann (2018) is of a fundamental nature, and is not affected by the amounts of training data or how expressive the learning system is.

## AI in psychiatry: some insights

Useful insights on how to achieve our goals can come from a review of recent work applying AI to psychiatry. In fact, AI can also help psychiatry, leading to the potential for a virtuous cycle. In an informative recent survey, Shatte, Hutchinson, and Teague (2019) summarised the methods and applications of ML in mental health and found that four main application domains emerged: detection and diagnosis, prognosis treatment and support, public health, and research and clinical administration. They concluded that there is still significant room to apply ML to other areas of psychology and mental health beyond the detection and diagnosis on which most ML studies focus. Durstewitz, Koppe and Meyer-Lindenberg (2019), in particular, reviewed the use of DL in psychiatry, with the motivation that DL algorithms often outperform previous ML methods in many applications, suggesting they may provide similar gains in psychiatry. They found that DL has mostly been applied to diagnostics in psychiatry, achieving convincing preliminary results. However, the small number of such studies does not yet support any firm conclusions. Durstewitz et al. (2019) also discussed some challenges for DL in psychiatry, such as low sample sizes, and some promising areas for their future development, such as interpretability, potentially through more biologically or cognitively inspired algorithms.

Autonomous robots have also recently been deployed in homes, to work with children with autism, and have been shown to lead to improvements in their math and social skills, through game personalisation and engagement modelling from behavioural cues (Jain, Thiagarajan, Shi, Clabaugh, & Matarić, 2020). The researchers involved in this project are now planning to extend the modelling to children's cognitive-affective states (ToM), including emotions such as confusion or excitement (Dawson, 2020).

Overall, however, we are not yet in a position to facilitate effective human–robot companionship for the treatment of psychiatric disorders.

## Machine Theory of Mind: a proposal

One possible way forward, which we support here, is to leverage on the successes of deep and RL not by attempting to learn in a brute-force fashion mappings from raw inputs (e.g. video or speech) to human intentions and reasoning processes, but by using them as tools to build suitable artificial ToM structures mimicking the actual behaviour of the human mind, in cross-disciplinary work incorporating neuroscientific insights and evidence.

Humans can predict others' mental states and actions by predicting how they themselves would act in a given situation – a mechanism called ‘internal simulation’. Similar simulations also have a role in other well-known mental functions, such as episodic and autobiographical memory, counterfactual thinking and episodic future thinking. In direct reference to the concept of hot cognition, internal simulation is much related to empathy, as a mechanism allowing us to better understand others. Brain structures called ‘mirror neurons’ appear to support this mental function, for they are activated both when an individual actually performs an action and when they see someone else doing the same (Gallese & Goldman, 1998).

In another relevant process, humans put other people into ‘stereotypical’ categories characterised by rough personality traits, in order to speculate what they might do. Indeed, the ventral and dorsal regions of the medial prefrontal cortex specialise in responding to elements related to the ‘self’ and to the ‘other’ (Mitchell, Macrae, & Banaji, 2006) and their patterns of activation seem to adapt to who is performing the action we observe.

A computational approach for modelling other agents' reasoning processes can then be outlined, which is centred on flexibly generating ToM simulations for specific classes of agents and contexts. As it happens in the human brain, such simulations should not be built from scratch for any new class of agent, but should result from assembling in different ways a number of basic ‘blocks’ (Andreas, Rohrbach, Darrell, & Klein, 2016). Such blocks would be deep neural networks representing logical relations between mental states (Liu et al., 2016). For example, if I observe an angry person, I may predict that they would be likely to hurt someone. The best way to connect these blocks to form the ‘best’ possible simulation for the observed scene and involved agents would then be learned from experience. For example, in a hallway where taking stairs or taking the lift are both options, we might infer through such a bespoke simulation that an elderly, frail person would take the lift, while a healthy young adult might take the stairs. This may be done according to the principles of RL (Mnih et al., 2015), by rewarding structures which lead to predictions that accurately match the observed behaviour, and by penalising those that lead to inaccurate ones.

## The way ahead

Significant obstacles remain on the way to an effective, neuroscience-inspired approach to machine ToM.

In human beings, simulations of other people's mental processes are slowly built over the years, as we go about living our lives. The amount of data required to build the sought flexible mechanism for constructing agent-specific ToM simulations is likely to be large. However, we believe the concept is mature for proof in at least relatively narrow domains of application, such as autonomous driving, in which humans only use a fraction of their cognitive capabilities and their possible intentions are limited to a small number of cases (e.g. ‘Should I turn right or stop?’). It is reasonable to expect that the amount of data needed should be smaller than that required by the naïve approaches discussed above.

The human brain is remarkably versatile: it can process and compute a thousand different tasks, as we learn from each of them and transfer knowledge between them. When driving, for instance, we make use of knowledge gathered while playing, say, with our nephews and nieces, knowledge which allows us to create a good general model of children's patterns of behaviour. In this sense, an effective, human-inspired machine ToM needs to emerge from machines learning to deal with multiple, loosely correlated tasks, rather than focusing on a simple, narrow objective.

Empirical validation is also an open question. Whereas multiple datasets designed for testing observed human behaviour in videos exist, there simply is no benchmark for assessing an algorithm's ToM abilities. Arguably, mental state annotations will need to be provided to data capturing the observed behaviour of people, describing, for instance, the emotional state, desire and intention of the various agents involved at any given time. This is likely to be very time-consuming, and to require the assistance of experts from neuroscience, cognitive psychology and biological psychiatry.

Last but not least, suitable measures of performance for assessing the quality of the inferred mental states will also need to be devised. One possibility is to assess the accuracy of ToM predictions on the observed behaviour of the agents, using measures commonly accepted in ML such as precision and recall. The alternative is to directly compare machine-generated mental state attributions with human guesses in combined, ground-breaking cross-disciplinary tests.

This reinforces our opinion that there is a strong need for those in the field of psychological medicine to contribute to the development of AI. This will not only facilitate enhanced human–machine interaction in healthcare, including in the area of mental health, but should promote a much needed ethical approach in AI.
